# Asperulosidic acid inhibits the PI3K/Akt/NF-κB pathway to suppress endotoxin-induced uveitis

**DOI:** 10.3389/fmed.2024.1524779

**Published:** 2025-01-07

**Authors:** Yong Du, Jing Lu, Lujia Feng, Long Zhao, Ping Wu, Yuxia He, Linbin Zhou, Xing Wang, Hui Peng

**Affiliations:** ^1^Department of Ophthalmology, Chongqing Key Laboratory for the Prevention and Treatment of Major Blinding Eye Diseases, The First Affiliated Hospital of Chongqing Medical University, Chongqing, China; ^2^Shenzhen Eye Hospital, Shenzhen Eye Institute, Jinan University, Shenzhen, China; ^3^State Key Laboratory of Ophthalmology, Zhongshan Ophthalmic Center, Sun Yat-sen University, Guangdong Provincial Key Laboratory of Ophthalmology and Visual Science, Guangdong Provincial Clinical Research Center for Ocular Diseases, Guangzhou, China

**Keywords:** uveitis, asperulosidic acid, EIU, inflammation, NF-κB

## Abstract

**Introduction:**

Uveitis, a severe inflammatory disease affecting the uvea, is associated with visual impairment and irreversible blindness. Asperulosidic Acid (ASPA), derived from *Hedyotis diffusa*, is known for its notable anti-inflammatory and antioxidant characteristics.

**Methods:**

The present study explored the potential anti-inflammatory effects and the fundamental processes of ASPA by injecting it or a placebo into the vitreous of rats with endotoxin-induced uveitis (EIU). The severity of the disease was assessed using clinical scores obtained through slit lamp examination. The study involved the examination of protein concentrations and cell count in the aqueous humor (AqH), the detection of inflammatory mediators expressed in the retina. We evaluated the expression levels of various proteins, including the tight junction protein ZO-1, the endothelial marker VE-cadherin, and the key inflammatory mediators NF-κB and its phosphorylated form, along with the regulatory proteins IκB-a and IKK in their phosphorylated and non-phosphorylated states.

**Results:**

ASPA treatment significantly reduced the clinical score of EIU, including inflammatory leukocyte penetration, protein accumulation, cellulose-like exudates, the expression of ICAM-1, IL-6, MCP-1, and TNF-α in the AqH; and adhesion of leukocytes. The activation of the PI3K/Akt/NF-κB pathway was observed in EIU. Nevertheless, pretreatment with ASPA significantly suppressed the release of ICAM-1, TNF-α, MCP-1, and IL-6.

**Discussion:**

ASPA may play a role in suppressing LPS-induced inflammation by obstructing the activation of the PI3K/Akt/NF-κB signaling pathway. As a result, ASPA has shown the capacity to significantly reduce immune inflammation.

## 1 Introduction

Uveitis is a research hotspot in recent years (1). Two thirds of patients with uveitis, a debilitating intraocular inflammatory illness, get permanent vision loss (2). It accounts for roughly 5–10% of worldwide instances of visual impairment as a result of its secondary complications and recurrence (3), including edema of the macula (4), lens opacities (5), and glaucoma (6). Uveitis primarily develops and arises as a consequence of inflammation (7), which has a complex etiology and is closely related to bacteria, viruses, and autoimmune diseases (8). Currently, there is no unified standard treatment for uveitis, and glucocorticoid therapy is commonly used clinically to reduce the severity and symptoms of eye inflammation (9). However, significant adverse effects can occur with prolonged exposure to these drugs (10, 11). Furthermore, even with intensive medical intervention, the possibility of pharmacological unresponsiveness and ongoing recurrence remains, potentially leading to severe outcomes like irreversible vision loss and total vision deprivation (2). There is an urgent need for innovative, effective and safe therapies.

Endotoxin-induced uveitis (EIU), alternatively referred to as lipopolysaccharide-induced ocular inflammation, serves as a well-accepted animal model for human uveitis, exhibiting pronounced pathological alterations such as protein leakage and the presence of inflammatory cells at the inflammation’s peak. In addition to the inflammatory reaction in the anterior segment, the retina and choroid are also involved in the pathological process (12, 13). The EIU model is widely used in the study of the pathogenesis and drug treatment of human uveitis due to its advantages of simplicity, high repeatability, and rapid modeling. The EIU animal model is created by injecting lipopolysaccharides from Gram-negative bacteria into the footpads, triggering a systemic inflammatory response and thus inducing acute experimental uveitis (14). The culmination of severe inflammatory responses is typically detected around 24 h after the injection of LPS. EIU is characterized by white blood cell attachment, damage or demise of retinal cells, and protein leakage into the eye (15, 16). A diverse array of cytokines, including IL-6, IL-4, IL-2, and IL-1, as well as intercellular adhesion molecule 1 (ICAM1), monocyte chemoattractant protein-1 (MCP-1), and tumor necrosis factor-α (TNF-α), are considered to be involved in the pathogenesis of uveitis (17–21). Therefore, exploring the regulatory mechanism of EIU cytokines is helpful to further explore the treatment of ocular inflammation.

Asperulosidic acid (ASPA), one of the 171 identified compounds, is a type of cyclic enol ether-derived terpene. It is sourced from *Hedyotis diffusa*, a plant that is common throughout China, Indonesia, and several other Asian regions (22, 23). Widely acknowledged for its therapeutic effects, ASPA exhibits anti-inflammatory and anti-tumor capabilities (24, 25). Studies in cellular and animal models have revealed that ASPA effectively hinders the inflammatory process by limiting the release and dispersal of cytokines (24, 26). Moreover, ASPA can notably decrease the rate of growth and the ability to move in esophageal cancer cells, thereby diminishing the tumor’s resistance to 5-fluorouracil by curbing the expression of cyclin-dependent kinase 2 (CDK2) and E2F1 (25). In a gestational diabetes mellitus mouse model, recent research has indicated that ASPA treatment markedly decreases both inflammation and oxidative stress (27). Taken together, these studies suggested that ASPA may serve as a potent anti-inflammatory drug candidate. However, its potential effect on inflammatory processes during the development of EIU remains uninvestigated. Consequently, it is important to use EIU model to study the effect of ASPA treatment on EIU.

In our research, we assessed the impact of ASPA on EIU within a living organism to reveal its effects and corresponding signaling pathways. Our findings revealed that administering ASPA to a rat model of EIU notably reduced clinical symptoms, including a significant decrease in protein levels, inflammatory cell infiltration, cellulose-like exudates, and pro-inflammatory cytokine production in the aqueous humor (AqH), as well as leukocyte adhesion. The onset of EIU has been associated with the observation of the PI3K/Akt/NF-κB signaling cascade’s activation. The intravitreal administration of ASPA was found to effectively diminish the synthesis of pro-inflammatory cytokines, consequently mitigating the inflammatory response by modulating this signaling network.

## 2 Materials and methods

### 2.1 Antibodies and reagents

The primary antibodies utilized in the study included anti-TNF-α (ab236712 and ab307164), anti-IL-6 (RAB0311 and ab9324), anti-MCP-1 (ab219045, ab214819 and ab25124), anti-ICAM-1 (ab100763 and ab282575), anti-NF-κBp65 (ab16502), anti-p-NF-κB (3033, Cell Signaling Technology, United States), anti-IKK (ab178872), anti-p-IKK (2697, Cell Signaling Technology, United States), anti-IκB-a (ab133462), anti-p-IκB-a (2859, Cell Signaling Technology, United States), anti-PI3Kp85 (ab182651), anti-pPI3Kp85 (abY607), anti-Akt (ab38449), and anti-p-Akt (abT308). Additionally, ASPA (HY-N6246, MedChemExpress) and LPS (Sigma-Aldrich, St. Louis, MO, United States) were procured from the specified sources.

### 2.2 Animals

Our experimental animals were male Sprague Dawley rats, specifically chosen for their weight (150–180 grams) and age (6–8 weeks), and were acquired from the Animal Experiment Center at Chongqing Medical University. The study was conducted with the approval of the Ethics Committee of Chongqing Medical University’s First Affiliated Hospital. Sodium pentobarbital was used to induce anesthesia in the rats at a rate of 350 mg/kg, intraperitoneally (i.p.). Following this, ASPA was administered in different doses through various routes: intraperitoneal, subconjunctival, and intravitreal. EIU was induced by injecting 200 μg of LPS into the footpad. The tissues were retrieved 24 h post the LPS injection for further examination. Compliance with the IACUC of NUCM’s institutional guidelines and the ARRIVE guidelines was a priority throughout the execution of all experimental procedures.

### 2.3 Intravitreal injection and subconjunctival injection

Rats were anesthetized with pentobarbital via intraperitoneal injection, and their pupils were dilated with the use of 0.5% of tropicamide. The eyelid was opened, and a tunnel, which began at a point that was 2 mm away from corneal limbus, was made by a 30-gauge beveled-needle (7803-07, Hamilton, Switzerland). A volume of 5 μL ASPA solution was administered into the vitreous cavity via the tunnel. A volume of 5 μL of a 0.1 mg/mL ASPA solution was administered into the conjunctival sac of the rats. Following the injection, antibiotic eye ointment was used to avert infection.

### 2.4 Uveitis grading

The slit lamp (Shangbang, Chongqing, China) was used to assess the presence of inflammation in the anterior chamber. Two observers conducted examinations 24 h after LPS injection, and the severity of uveitis was evaluated based on Hoekzema’s scoring system as described in Table 1.

**Table 1:** Scoring system for clinical evaluation of uveitis.

Clinical signs	Grade of uveitis (score)
Iris hyperemia	
Absent	0
Mild	1
Moderate	2
Severe	3
Pupil	
Normal	0
Synechia	1
Exudate in anterior chamber	
Absent	0
Small	1
Large	2
Hypopyon	
Absent	0
Present	1
Maximum possible score	7

### 2.5 Histology

Post-LPS administration for 24 h, six rats were deliberately selected for humane sacrifice through the intraperitoneal administration of sodium pentobarbital at a dosage sufficient to ensure a painless death. After the 12-h fixation in FAS fixative, the rat eyeballs were then embedded in paraffin, and after the paraffin block had hardened, 5 μm thick sections were sectioned along the vertical midline of the optic nerve. The sections were then dewaxed and rehydrated before being stained with hematoxylin and eosin.

### 2.6 Cell count and protein concentration

Using a 30-gauge needle, the researchers executed an anterior chamber puncture to both enumerate cells and determine the concentrations of proteins present in the aqueous humor of the rats. Cell counts were manually enumerated using a hemocytometer under a light microscope, and overall protein content was assessed using a BCA Protein Concentration Determination Kit (Beyotime Biotechnology, P0009).

### 2.7 Cell culture

The human RPE cell line, ARPE-19 that was purchased from Procell Life Science & Technology Co., Ltd. (CL-0026; China). In an incubator maintaining conditions of 37°C and 5% CO_2_, cells were cultivated in a full medium consisting of 89% RPMI-1640/DMEM high glucose, supplemented with 10% newborn bovine serum and 1% penicillin-streptomycin. The schedule for medium renewal, usually every 1–2 days, was governed by cellular growth patterns.

### 2.8 CCK-8 assay

Cell Counting Kit-8 reagent (C0038, Dojindo, Japan) and cell culture medium were mixed in a ratio of 1: 9 in darkness. Then, the cells were treated with the mixed CCK-8 reagent after the cells were washed twice softly with PBS. Two hours later, the absorbance of the liquid in each well was measured at the wavelength of 450 nm.

### 2.9 RT-PCR

The total mRNA was extracted using TRIzol Reagent (Invitrogen, 15596026). The process of cDNA creation from RNA was succeeded by RT-PCR, facilitated by the LightCycler 480 SYBR Green I Master (Roche, Indianapolis, IN, United States). Gene expression data were standardized to GAPDH or β-actin as reference genes, with the corresponding primer sequences outlined in Tables 2, 3.

**Table 2:** Sequences of PCR primers (*Rattus norvegicus*).

Gene	Forward sequence (5′–3′)	Reverse sequence (5′–3′)
*ICAM-1*	TCTGTGTCCCCCTCAAAAGTC	GGGGTCTCTATGCCCAACAA
*MCP-1*	CATAGCAG CCACCTTCATTCC	TCTCCTTGGCCA CAATGGTC
*TNF-α*	AACAAGGAGGAGAAGTTCCCAAA	CTCCTCCGCTTGGTGGTTT
*IL-6*	AGGATACCACCCACAACAGACC	TTGCCATTGCACAACTCTTTTC
*GAPDH*	TTCCTACCCCCAATGTATCCG	CATGAGGTCCACCACCCTGTT

**Table 3:** Sequences of PCR primers (*Homo sapiens*).

Gene	Forward sequence (5′–3′)	Reverse sequence (5′–3′)
*ICAM-1*	CCTCACCGTGTACTGGACTC	CAGTGCGGCACGAGAAATTG
*MCP-1*	CTCGCTCAGCCAGATGCAAT	TTGGGTTTGCTTGTCCAGGT
*TNF-α*	AGCCCATGTTGTAGCAAACC	GGCTCTTGATGGCAGAGAGG
*IL-6*	TCCTGGTGTTGCCTGCT	CACCAGGCAAGTCTCCTCAT
*β-actin*	ATTCCTATGTGGGCGACGAG	AAGGTCTCAAACATGATCTGGGT

### 2.10 Western blot analysis

After homogenization of the rat eyeball samples with a RIPA Protein Extraction Kit (Beyotime Biotechnology, P0013B), the protein concentration was quantified using a BCA Protein Assay Kit (Beyotime Biotechnology, P0009). The proteins were then resolved on a gel by SDS-PAGE and transferred to PVDF membranes for analysis (Millipore). The membranes underwent an hour of blocking at room temperature with a 5% skim milk solution, followed by overnight incubation with primary antibodies at 4°C. After the PBST wash, the membranes were incubated for an hour with horseradish peroxidase-conjugated secondary antibodies at ambient temperature. The subsequent luminescent reaction facilitated antibody detection, with care taken to keep within the ideal band detection limits. Each sample underwent three independent technical replicates.

### 2.11 Immunofluorescent staining

HRVEC cells were seeded onto Lab-Tek chamber slides at a concentration of 2 × 10^4^ cells/well. After confirming cell adhesion, the cells were initially activated with ASPA (200 μg/mL) for 1 h, subsequently, were challenged with LPS (50 ng/mL) for 24 h. A 10-min fixation with 4% PFA was followed by a 30-min blocking step with 10% BSA, after which the cells were exposed to ZO-1/VE-Cadherin antibodies overnight at a dilution ratio of 1:200. Incorporating a fluorescent tag, secondary antibodies were used to treat the cells for an hour at 37°C. The slides were stained with DAPI, mounted in glycerol, and then the images were captured with a fluorescence microscope (Leica K5, Heidelberg, Germany).

### 2.12 Statistical analysis

The statistical analysis utilized SPSS 26.0 software. Analyses of group relationships were conducted using an unpaired *t*-test and one-way ANOVA, with Tukey’s post-hoc test utilized to measure variability. Presented data reflect the mean ± standard error, obtained from no fewer than three individual tests, using a *p*-value threshold of less than 0.05 for statistical significance.

## 3 Results

### 3.1 ASPA treatment via intravitreal injection mitigates EIU

**Figure 1:**
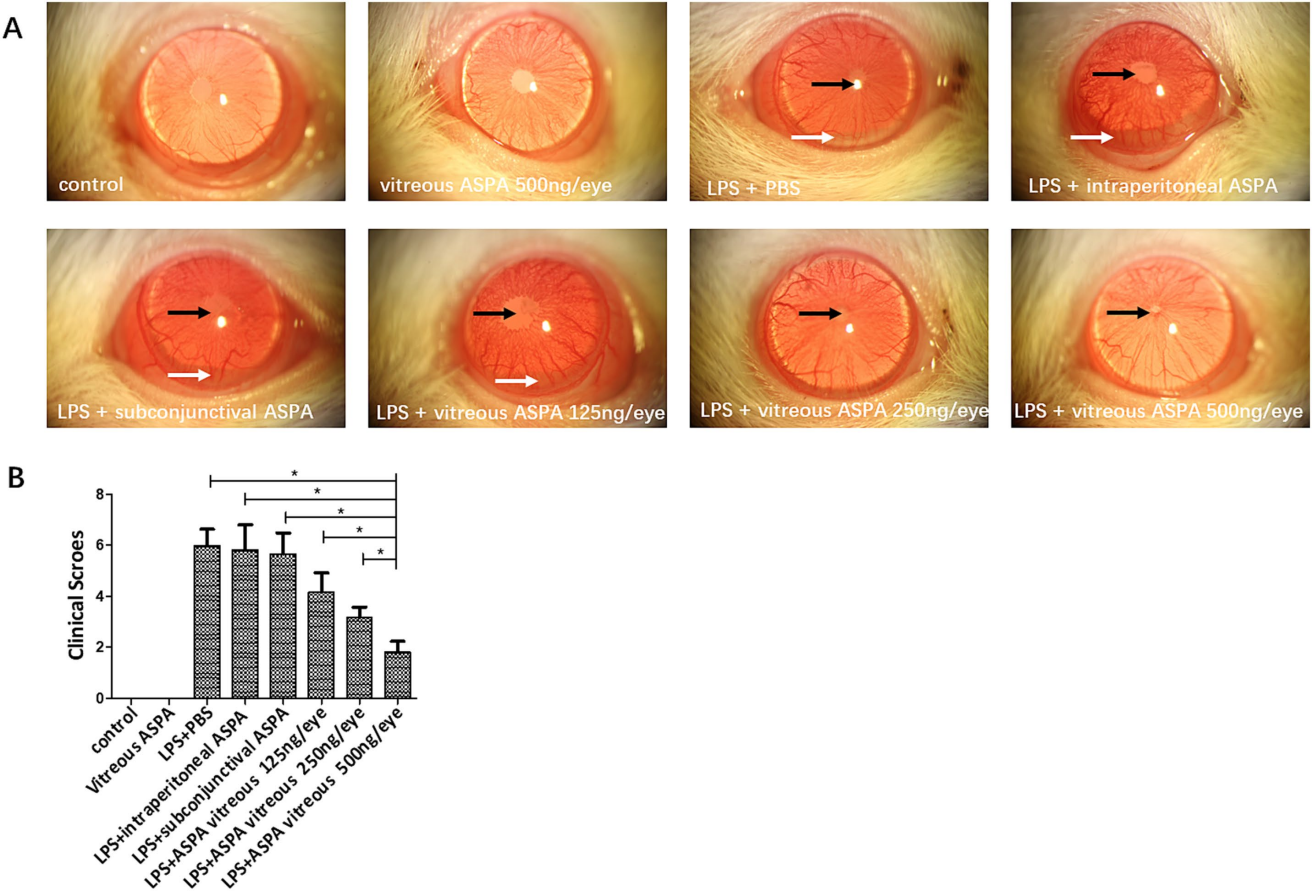
Intravitreal injection of ASPA treatment demonstrated a reduction in EIU. Rats were treated with ASPA 24 h before LPS inoculation through intraperitoneal, subconjunctival, and intravitreal routes at doses of 10, 30, and 100 μg/kg, and 125, 250, and 500 ng/eye, respectively. **(A)** A day prior to the LPS challenge, the LPS + PBS group exhibited pupil occlusion (black arrow), a fibrinous membrane (black arrow), and hypopyon (white arrow). No inhibitory impact on inflammation was observed in the groups receiving ASPA via intraperitoneal, subconjunctival, or 10 ng/eye intravitreal injection. At 24 h post-LPS injection, a slight fibrinous membrane formation and iris blood vessel dilation were noted in the 250 and 500 ng/eye intravitreal ASPA groups, indicating a dose-dependent treatment effect. Cular inflammation was absent in rats from both the control and the ASPA-injected intravitreal groups when LPS was not introduced. Magnification: 16×. **(B)** Clinical scores were conducted 24 h following the administration of LPS. Clinical scores of the eight distinct groups are depicted in a histogram, where the values are expressed as the mean ± standard deviation, with six eyes per group (*n* = 6). ^*^p < 0.05 significantly different.

The therapeutic potential of ASPA on EIU was examined by administering it to rats via intraperitoneal injection at varying doses (10, 30, and 100 μg/kg), and also through subconjunctival and intravitreal routes at doses of 125, 250, and 500 ng/eye, a day before LPS challenge. After 24 h of LPS injection, rats exhibited ciliary artery congestion, dilation of the iris vasculature, development of a fibrinogen membrane, obstruction of the pupil, and the presence of hypopyon. Inflammation scores, indicative of clinical signs, were evaluated a full day post the administration of LPS (Table 1). The results indicated that intravitreal ASPA injection significantly alleviated ocular symptoms in EIU rats and reduced clinical inflammatory scores (Figures 1A,B). However, neither intraperitoneal nor subconjunctival injection of ASPA at the aforementioned doses showed any relief of inflammation in EIU rats, and the clinical inflammatory scores did not exhibit any notable variations, indicating a lack of substantial impact on the observed parameters (Figures 1A,B).

In summary, intravitreal injection of ASPA can significantly alleviate inflammation in EIU rats, and this administration method was adopted in subsequent experiments. Figures 1A,B indicate that intravitreal injection of ASPA (500 ng/eye) significantly alleviated LPS-induced ciliary artery congestion, iris vasodilation, development of a fibrinogen membrane, obstruction of the pupil, and the presence of hypopyon in EIU rats. All these results indicate that treatment with intravitreal injection of ASPA can alleviate inflammation in EIU rats.

### 3.2 ASPA suppresses inflammation in EIU

**Figure 2:**
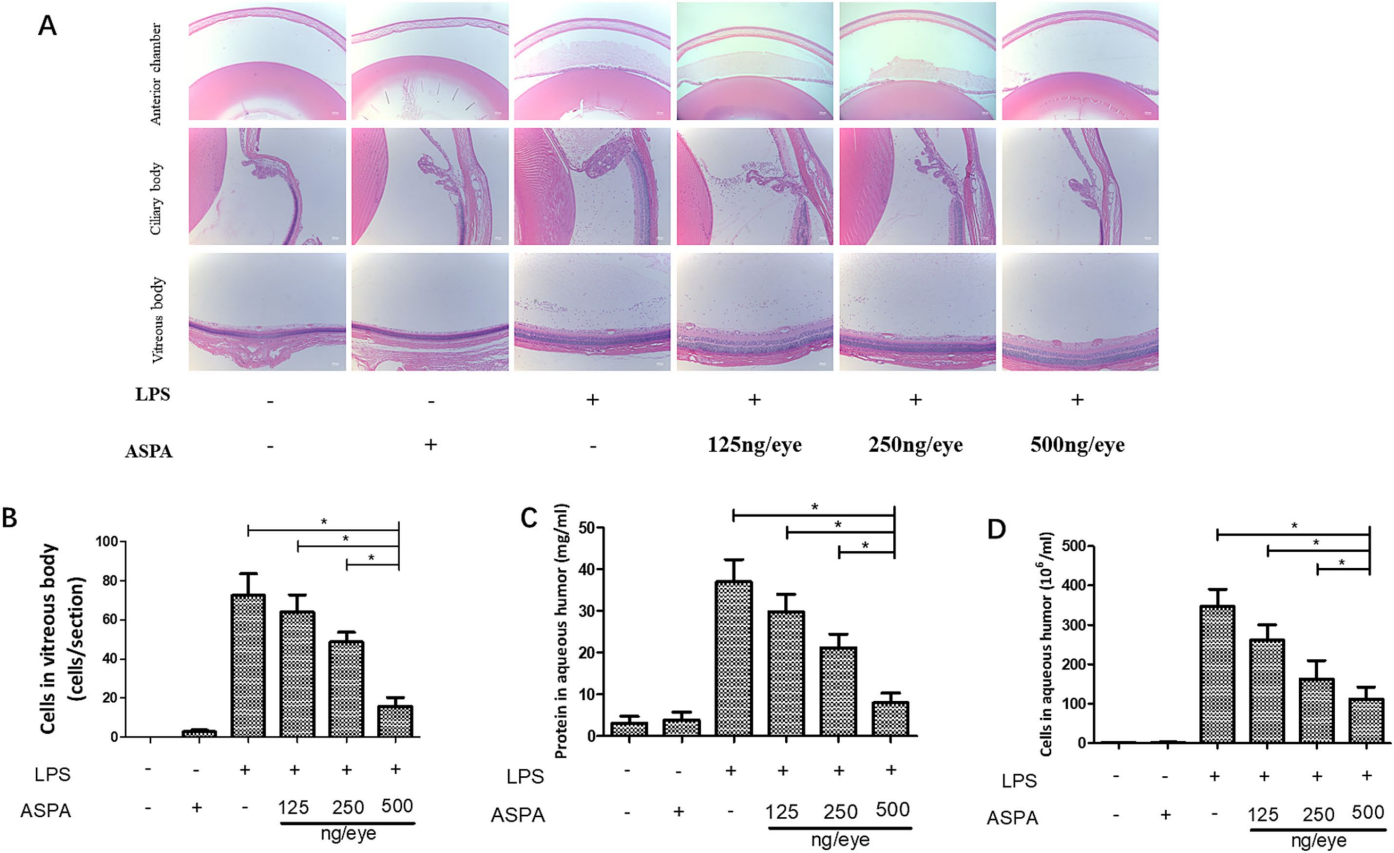
ASPA administration effectively reduces inflammation in EIU. Rats received intravitreal injections of ASPA at doses of 125, 250, and 500 ng/eye 24 h prior to LPS injection. Tissue samples were obtained 24 h after LPS treatment. At the 24-h mark post-LPS injection, a significant infiltration of inflammatory cells, indicated by black arrows, was detected in the anterior chamber, iris stroma, ciliary body, and vitreous body of the LPS + PBS group. **(A)** The intravitreal administration of ASPA at a dosage of 125 ng/eye failed to demonstrate any suppression of inflammation. **(B)** In the AqH, a considerable cell count was detected 24 h following LPS injection in both the LPS + PBS group and the group that received 125 ng/eye intravitreal injections. A notable reduction in AqH cell density was noted in the groups treated with 250 and 500 ng/eye of intravitreal ASPA, which was found to be dose-dependent. The scale bar is 50 μm. **(C)** The protein concentrations in the front chamber were measured across six separate trials. **(D)** The number of inflammatory cells in vitreous body. All the data are shown as means ± standard deviation (*n* = 6, eye). ^*^p < 0.05 significantly different.

Our study explored the impact of intravitreal ASPA injection on curtailing the inflammatory processes characteristic in EIU. In the context of EIU, histological examination evidenced an accumulation of inflammatory exudates within the anterior chamber. Conversely, the application of ASPA treatment notably diminished these exudative accumulations (Figure 2A). Moreover, EIU caused inflammatory cell infiltration in the ciliary body, which was significantly reduced by intravitreal ASPA treatment (Figure 2B). In the vitreous body, EIU triggered a noticeable infiltration of inflammatory cells. In contrast, ASPA treatment markedly decreased this cellular penetration when juxtaposed with untreated control subjects, with the therapeutic impact depending on the dosage (Figure 2D). he presence of EIU, in comparison to the control group, caused a pronounced upsurge in the AqH’s cellular and protein densities. In contrast, administration of ASPA markedly decreased the concentrations of proteins in the AqH of rats subjected to treatment. The efficacy of the treatment was observed to be dose-dependent, as depicted in Figure 2C. ASPA led a significant dose-dependent reduction in the quantity of inflammatory cells present in the anterior chamber of EIU rats (Supplementary Figure S1). These findings suggest that ASPA mitigates EIU in rats by effectively inhibiting the inflammatory response.

### 3.3 ASPA improved cell connectivity reduction caused by LPS

**Figure 3:**
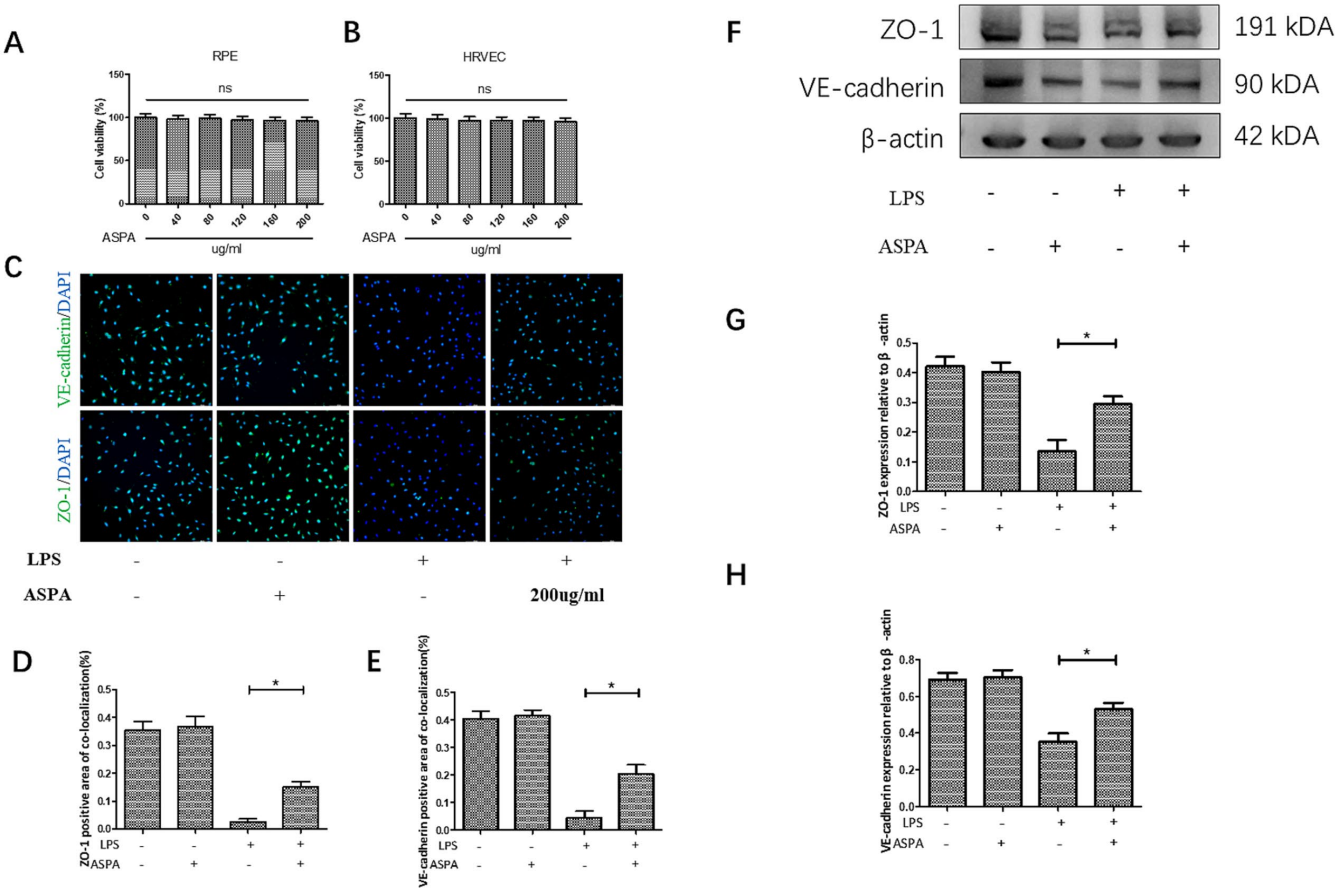
ASPA improved cell connectivity reduction caused by LPS. CCK-8 assay of cell viability of RPE **(A)** and HRVEC **(B)**. **(C)** In HRVEC cells, the immunofluorescence assay demonstrated a marked decrease in the expression levels of ZO-1 and VE-cadherin following LPS treatment. The scale bar is 100 μm. ASPA at the concentration of 200 μg/mL could reverse the decrease in the expression induced by LPS **(D,E)**. The expression levels of ZO-1 and VE-cadherin were assessed using the western blot technique **(F)**. ASPA effectively counteracted the LPS-induced reduction in the expression of ZO-1 **(G)** and VE-cadherin **(H)**. All the data are shown as means ± standard deviation (*n* = 3). ^*^p < 0.05 significantly different.

Utilizing the CCK-8 assay (Figures 3A,B), we assessed the cell viability and found that a concentration of 200 μg/mL did not affect the metabolic activity of both retinal pigment epithelial cells (ARPE19) and human retinal vascular endothelial cells (HRVEC). It has been confirmed that LPS can decrease the expression of proteins related to the maintenance of tight junctions in endothelial cells, thereby enhancing the permeability of these cells. This reduction in tight junctions between endothelial cells will further exacerbate the infiltration of inflammatory cells into tissues. The immunofluorescence technique demonstrated that LPS activation in HRVECs caused a pronounced reduction of the tight junction proteins ZO-1 and VE-cadherin (Figures 3C–E). However, treatment with ASPA at a concentration of 200 μg/mL effectively reversed the downregulation induced by LPS. Similar findings were confirmed through western blot analysis (Figures 3F–H). Similarly, we obtained the retina of EIU rats for western blot (Supplementary Figure S2A) detection and found that ASPA (500 ng/eye) intravitreal injection significantly reduced the expression of ZO-1 (Supplementary Figure S2B) and VE-cadherin (Supplementary Figure S2C).

### 3.4 ASPA reduced the production of proinflammatory mediators *in vitro*

**Figure 4:**
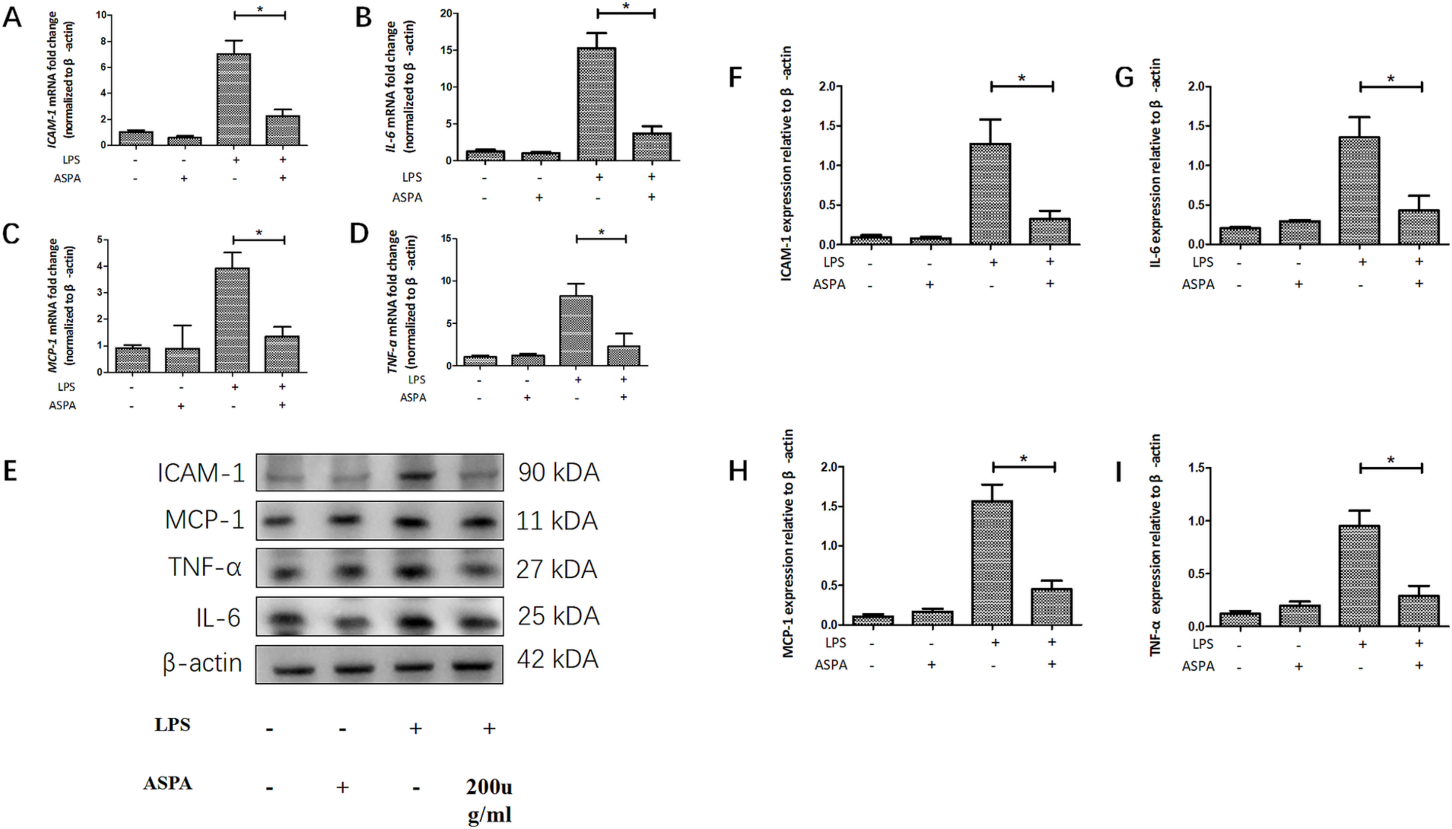
ASPA decreased the release of proinflammatory mediators *in vitro*. RPE cells were pretreated with 200 μg/mL ASPA for 1 h before 50 ng/mL LPS stimulated RPE cells for 24 h. **(A–D)** RT-PCR served as the method for assessing the transcriptional levels of proinflammatory proteins ICAM-1, IL-6, MCP-1, and TNF-α. **(E–I)** Western blot was employed to assess the presence of ICAM-1, IL-6, MCP-1, and TNF-α in the samples. All the data are shown as means ± standard deviation (*n* = 3). ^*^p < 0.05 significantly different.

To confirm the inhibitory effect of ASPA in vitro, RPE cells were exposed to 200 μg/mL ASPA for 1 h, followed by stimulation with 50 ng/mL LPS for 24 h. Subsequently, the levels of inflammatory factor mRNA and proteins were extracted and analyzed in RPE cells. LPS was found to induce a substantial upregulation of mRNA (Figures 4A–D) and protein (Figures 4E–I) levels for *ICAM-1*, *TNF-α*, *MCP-1*, and *IL-6* in RPE cells. Furthermore, ASPA at a concentration of 200 μg/mL significantly mitigated the heightened expression of these inflammatory cytokines induced by LPS. These results indicate that ASPA exerts a notable suppressive impact on the inflammation of RPE cells triggered by LPS.

### 3.5 ASPA reduced the *in vivo* production of proinflammatory mediators

**Figure 5:**
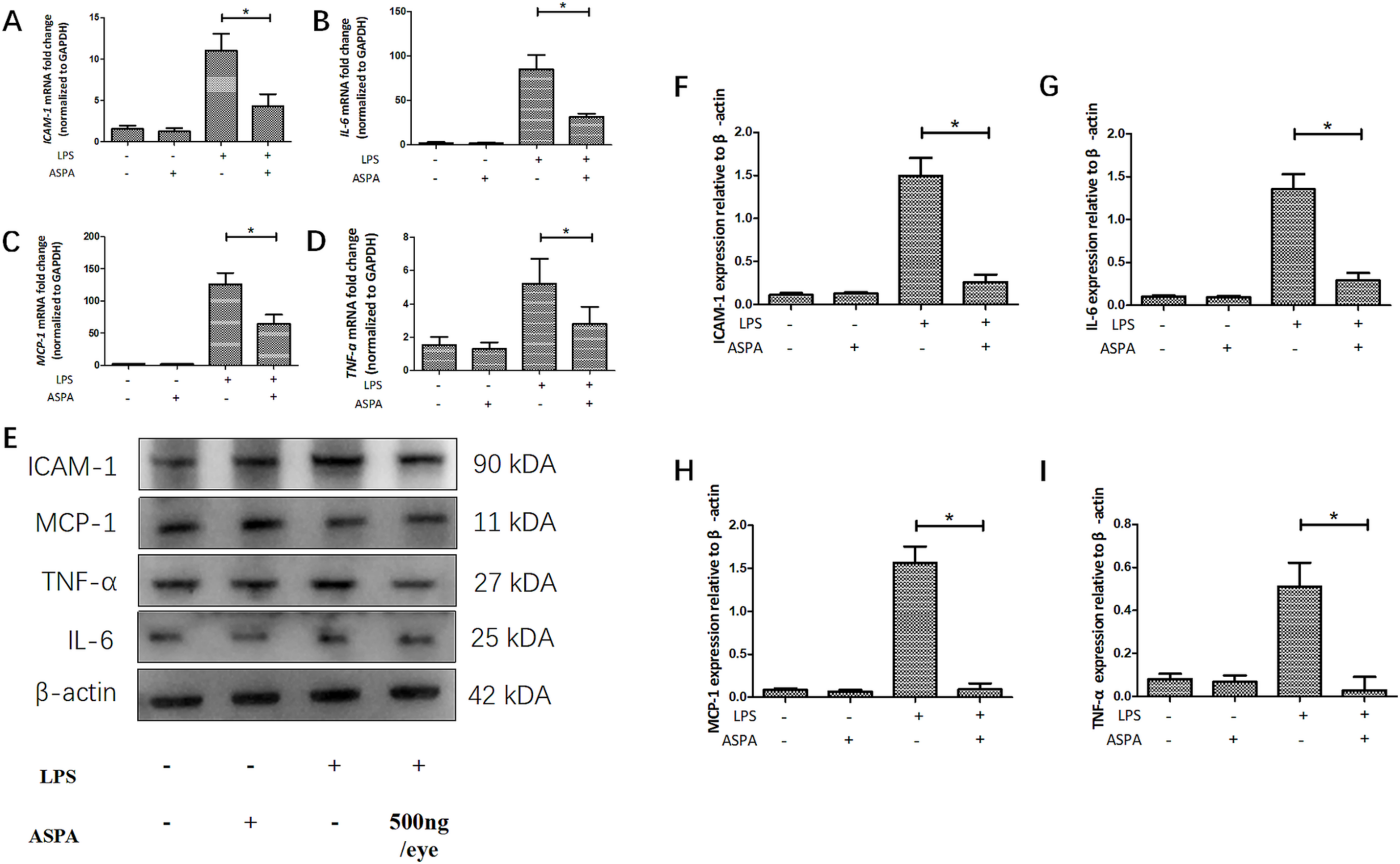
ASPA reduced the production of proinflammatory mediators *in vivo*. Before the injection of LPS, ASPA (500 ng/eye) was administered into the vitreous cavity of rats 24 h prior. Tissues were collected 24 h post-LPS administration. **(A–D)** Our analysis of ICAM-1, IL-6, MCP-1, and TNF-α expression levels was conducted using the RT-PCR. **(E–I)** Western blot was the chosen method for assessing the levels of the inflammatory markers ICAM-1, IL-6, MCP-1, and TNF-α. The data are presented as mean ± standard deviation (*n* = 6, eye). ^*^p < 0.05 significant difference.

After LPS administration, a significant surge in proinflammatory factors, especially ICAM-1, IL-6, MCP-1, and TNF-α, is observed in EIU, reaching its peak around 24 h post-treatment. In order to understand the mechanism by which ASPA inhibits inflammation in EIU, we evaluated the levels of specific inflammatory markers, including ICAM-1, IL-6, MCP-1, and TNF-α. The commencement of EIU led to an increase in multiple inflammatory markers, such as *ICAM-1*, *IL-6*, *MCP-1*, and *TNF-α*, as indicated by our RT-PCR findings. Post-ASPA administration, there was a considerable decline in the mRNA expression of inflammatory markers *ICAM-1*, *IL-6*, *MCP-1*, and *TNF-α* (Figures 5A–D). Additionally, western blot analysis revealed a marked decrease in protein levels of ICAM-l (Figure 5F), IL-6 (Figure 5G), MCP-l (Figure 5H) and TNF-α (Figure 5I) after ASPA treatment as depicted in Figure 5E. These results indicate that ASPA effectively attenuated inflammation by downregulating pro-inflammatory mediators.

### 3.6 ASPA inhibits RPE cells inflammation through the PI3K/Akt/NF-κB pathway

**Figure 6:**
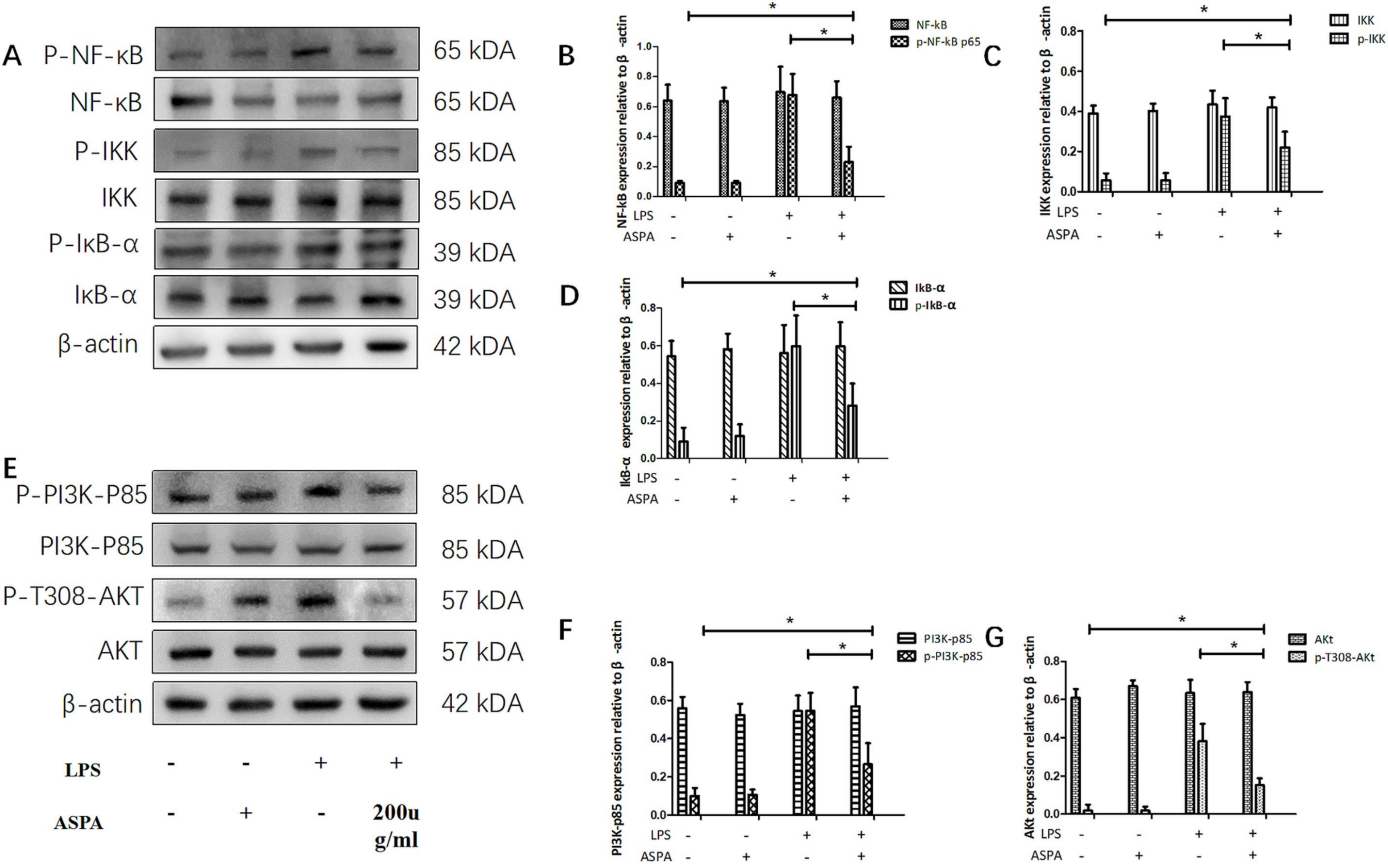
ASPA inhibits RPE cells inflammation through the PI3K/Akt/NF-κB pathway. RPE cells were pretreated with 200 μg/mL ASPA for 1 h before 50 ng/mL LPS stimulated RPE cells for 24 h. Western blotting **(A,E)** was utilized to examine the levels of signaling proteins, including PI3K-p85, p-PI3K-p85 **(F)**, Akt, p-T308-Akt **(G)**, NF-κB, p-NF-κBp65 **(B)**, IKK, p-IKK **(C)**, IκB-α, and p-IκB-α **(D)**. The data are presented as mean ± standard deviation (*n* = 3). ^*^p < 0.05 significantly different.

With the aim of uncovering the underlying mechanism of ASPA inhibiting RPE cell inflammation induced by LPS. RPE cells were pretreated with 200 μg/mL ASPA for 1 h before 50 ng/mL LPS stimulated RPE cells for 24 h. We performed western blotting to analyze NF-κB, p-NF-κB, IKK, p-IKK, IκB-α, p-IκB-α, PI3Kp85, p-PI3K-p85, Akt, and p-T308-Akt. LPS treatment, as indicated by our findings, led to an upregulation in the expression levels of p-NF-κB, p-IKK, p-IκB-α, p-PI3K-p85 and p-T308-Akt in RPE cells. However, ASPA treatment significantly inhibited this expression (Figure 6), suggesting effective suppression of inflammation by ASPA targeting the PI3K/Akt/NF-κB pathway.

### 3.7 ASPA inhibits EIU through the PI3K/Akt/NF-κB pathway

**Figure 7:**
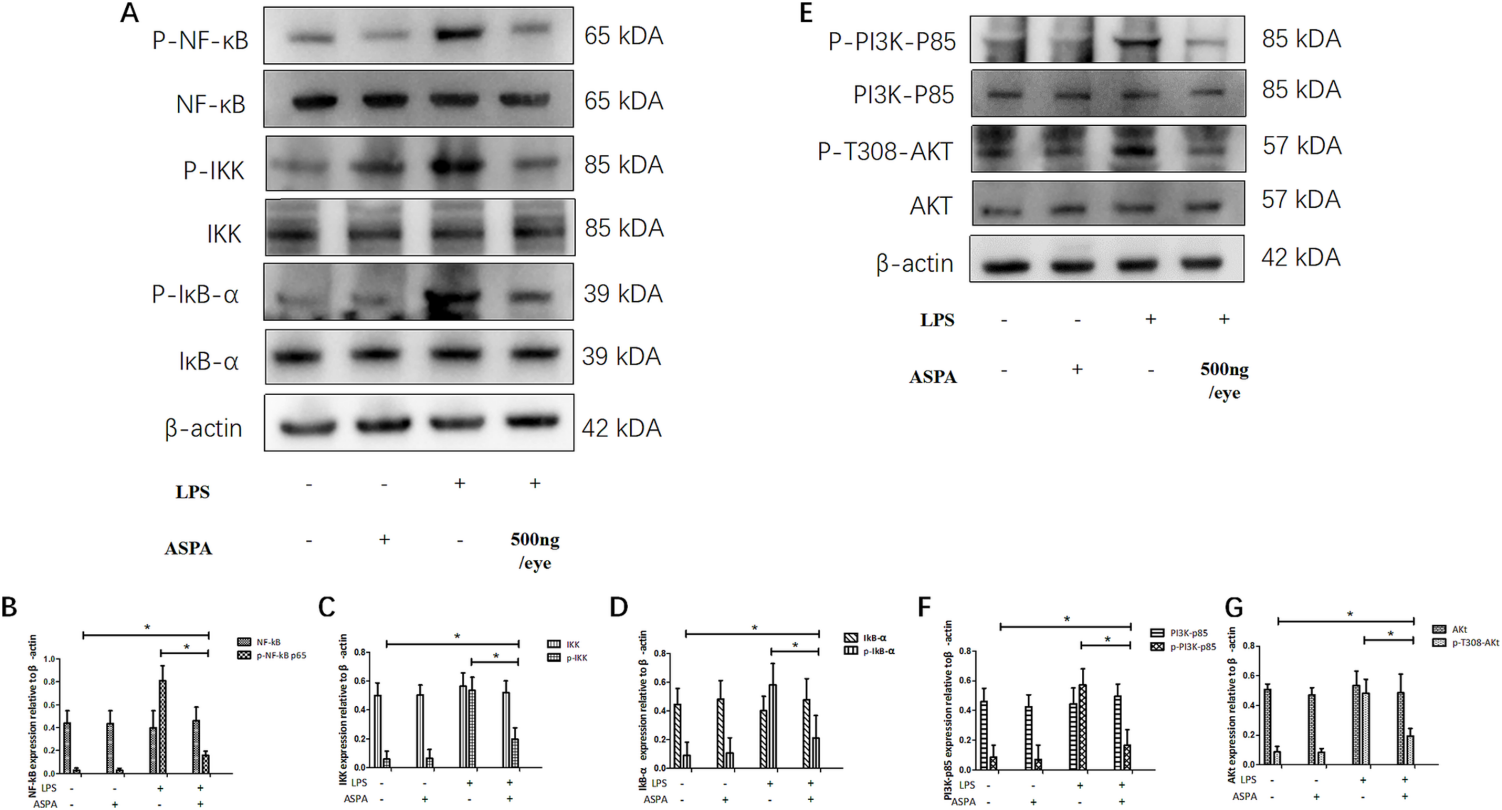
ASPA was found to have a suppressive effect on EIU by modulating the PI3K/Akt/NF-κB signaling pathway. In rat model, ASPA was administered into the vitreous cavity at a dosage of 500 ng/eye, 24 h prior to LPS injection. Western blot analysis **(A,E)** was employed to determine the expression levels of key signaling proteins, including PI3K-p85, p-PI3K-p85 **(F)**, Akt, p-T308-Akt **(G)**, NF-κB, p-NF-κBp65 **(B)**, IKK, p-IKK **(C)**, IκB-α, and p-IκB-α **(D)**, in tissue samples obtained 24 h after LPS administration. The data are presented as mean ± standard deviation (*n* = 6, eye). ^*^p < 0.05 significantly different.

NF-κB, a key regulatory protein, is integral to the orchestration of immune and inflammatory reactions, including the pathology of uveitis. We investigated the impact of ASPA on leukocyte adhesion by influencing NF-κB signaling. Our analysis revealed that ASPA treatment significantly reduced the elevated expression of p-NF-κB protein in the EIU group (Figure 7A). Furthermore, in the context of EIU, we investigated the mechanism by which ASPA controlled the activity of NF-κB through the manipulation of the IKK/IκB-α signaling pathway. WB results indicated that EIU induction induce a notable increase in p-IκB-α and p-IKK, while reducing IκB-α expression. Conversely, ASPA treatment effectively suppressed the expression of p-IκB-α and p-IKK, while increasing IκB-α expression (Figures 7B–D). These findings suggest that ASPA inhibits NF-κB activation by reducing IκB-α degradation through inhibition of the activated IKK complex induced by EIU. Our findings suggest that activation of the NF-κB pathway is implicated in the anti-inflammatory effects of ASPA in EIU.

Given the pivotal function of the PI3K/Akt pathway in leukocyte adhesion and NF-κB activation, our research subsequently sought to ascertain the involvement of this pathway in the anti-inflammatory effects of ASPA within EIU. To assess the levels of PI3Kp85, p-PI3K-p85, Akt, and p-T308-Akt, we performed western Blot analysis. While EIU caused an increase in the expression of p-T308-Akt and p-PI3K-p85, ASPA treatment notably decreased these levels in the EIU rat model (Figures 7E–G). Our study findings indicate that ASPA mitigated the inflammatory response in experimental autoimmune uveitis by influencing the activity of the PI3K/Akt/NF-κB signaling pathway.

## 4 Discussion

Uveitis represents a grave ocular affliction, distinguished by acute inflammation occurring inside the eye, and currently, there are several animal models used for uveitis research. EIU serves as an animal model for certain types of human uveitis, especially acute anterior uveitis. The Rubiaceae family is known to contain ASPA, which is distinguished by its bicyclic cis-fused cyclopentane-pyran molecular framework. Previous research has confirmed that ASPA possessed anti-oxidant and anti-inflammatory activities (23, 24). However, as of now, there is no research indicating whether ASPA has a therapeutic effect on uveitis. Our research focused on examining the impact of ASPA, at different dosages and via diverse administration pathways, on the treatment of uveitis in EIU rats. Our findings revealed that intraperitoneal and subconjunctival injection of ASPA did not demonstrate a significant therapeutic effect in EIU rats. Nevertheless, following the intravitreal administration of ASPA, in the context of EIU, a pronounced reduction in ocular inflammation was detected. In the context of EIU, ASPA effectively decreased the levels of inflammatory mediators including ICAM-1, IL-6, MCP-1, and TNF-α. Leukocytes are essential in the development of EIU, as MCP-1 facilitates their movement towards inflammation sites and ICAM-1 encourages their attachment. Furthermore, the function is regulated by IL-6 and TNF-α. The diminished transport of NF-κB (reflected by p-NF-κBp65) to the nucleus correlated with the anti-inflammatory outcomes, and this process was influenced by the PI3K/Akt pathway, ultimately leading to its decrease—a crucial step in deactivating inflammation. The results indicate ASPA’s potential as a therapeutic option for EIU, demonstrating its ability to suppress inflammation by interfering with the PI3K/Akt/NF-κB pathway.

There have been studies reporting that ASPA regulates inflammation in various disease models, while ASPA may exert diverse effects on various tissues within distinct pathological frameworks, these effects are not uniformly observed. According to previous studies, ASPA can protect renal function and inhibit fibrosis by anti-inflammatory and inhibition of NF-κB and TGF-β1/smad2/smad3 signaling pathways (23, 28). Wu et al. (27) found that ASP can reduce oxidative stress and inflammatory responses in the placenta of pregnant diabetic rats by regulating oxidative stress-related biomarkers. Importantly, in RAW 264.7 cells induced by LPS (24), ASPA significantly reduced the production of inflammatory factors. Consistent with previous studies, our study shows that ASPA treatment significantly reduces the levels of inflammatory responses and the concentrations of cytokines with pro-inflammatory properties triggered by EIU. However, when ASPA was injected into the peritoneal cavity and conjunctival sac of EIU rats, no significant therapeutic effects were observed. Nevertheless, the introduction of ASPA into the vitreous cavity resulted in a marked decrease in the ocular inflammation associated with EIU. Surprisingly, ASPA significantly improved the decreased level of VE-cadherin and ZO-1 in LPS-induced HRVEC, which may explain why ASPA can inhibit neutrophil infiltration and inflammatory cytokine levels in the eye.

Proinflammatory mediators like IL-6, TNF-α, MCP-1, and ICAM-1 play a key role in attracting neutrophils during inflammation and serve as important mediators of eye-related inflammatory responses (29). As a cell adhesion molecule within cells, the intraocular fluid of uveitis patients exhibited a higher percentage of CD4^+^ and CD8^+^ lymphocytes due to ICAM-1, in comparison to the control group (30). Becker et al. (31) have discovered that the use of ICAM-1 antibodies suppressed leukocyte adhesion and infiltration in murine EIU, while having no effect on rolling. Yoshida et al. (32) have discovered that levels of MCP-1 in the aqueous humor were notably elevated in patients with non-infectious or infectious uveitis compared to controls. It was demonstrated that knocking out MCP-1 inhibited the infiltration of inflammatory cells in the eye triggered by EIU (33). TNF-α, a cytokine that can be stimulated by signaling through Toll-like receptors or initiated by heightened concentrations of other cytokines, intensifies the degree of uveitis. The heightened presence of TNF-α encourages the development of T-cells towards proinflammatory subsets. Treatment with a TNF-α blocker exhibited positive effects on various manifestations of uveitis (34). IL-6 induces inflammation in the eyes, partly by promoting Th17 cell differentiation through IL-6 in uveitis (35). Injecting an anti-IL-6 antibody into the eye reduced experimental autoimmune uveitis in mice (36). The exact molecular mechanism through which ASPA inhibits inflammation remains incompletely understood. Previous studies have suggested that ASPA may alleviate placental oxidative stress and inflammatory responses by suppressing IL-6 and TNF-α, as well as regulating oxidative stress-related biomarkers (27). LPS-induced RAW 264.7 cells (24) exhibited a notable response to ASPA, characterized by a significant reduction in nitric oxide (NO) and prostaglandin E₂ (PGE₂) synthesis and decreased production of tumor necrosis factor-α (TNF-α) and interleukin-6 (IL-6). ASPA not only inhibited the activities of inducible nitric oxide synthase (iNOS) and cyclooxygenase-2 (COX-2) but also decreased the levels of *TNF-α* and *IL-6* mRNA. Echoing previous research, our investigation confirmed that EIU causes an overproduction of TNF-α and IL-6 and boosts the levels of *ICAM-1* and *MCP-1* at both the mRNA and protein levels. Nevertheless, ASPA treatment notably reduced their expression. Furthermore, we observed the presence of leukocytes in the eye’s anterior chamber, ciliary body, and vitreous body. Significantly, ASPA was observed to effectively inhibit leukocyte infiltration triggered by EIU in these three ocular locations for the first time. These results offer compelling evidence that ASPA suppresses inflammatory mediators and leukocyte activity, thereby preventing visual impairment associated with uveitis.

Current research on the detailed signaling pathways that ASPA uses to suppress inflammatory mediators is nascent; however, studies imply that its anti-inflammatory capabilities could stem from the inhibition of NF-κB and MAPK pathways (24, 27). In conditions like iritis, Vogt–Koyanagi–Harada (VKH), and Behçet’s syndrome—collectively categorized under uveitis—the NF-κB signaling pathways are known to contribute to disease severity (37). By sequestering NF-κB within the cellular cytoplasm, IκB inhibitor proteins effectively control and deactivate the NF-κB/Rel family of nuclear transcription factors. IκBs, once phosphorylated, are destined for dissociation, which in turn lifts their inhibitory influence on NF-κB (38). Activation of the IKK complex by cytokines prompts the phosphorylation of IκBs at particular locations, which then leads to the degradation of these proteins (39). A serine kinase has been identified through molecular cloning as a constituent of IKK. The IKK protein kinase, of particular interest, is integral to the control of NF-κB activation. In this research, we discovered a new finding that ASPA was able to efficiently decrease the breakdown of IκB-α by deactivating IKK, which became active during EIU initiation. The activation of NF-κB in EIU was suppressed by ASPA. It is worth noting that the PI3K/Akt pathway is vital to active NF-κB. LPS-induced IκB degradation was mitigated through the inhibition of PI3Kp85 and Akt phosphorylation, which consequently lowered the activation of the IKK complex. In peritoneal macrophages, the inhibition of the PI3K/Akt/IκB-α/NF-κB pathway, as shown by Huang et al. (40), led to a reduction in the production of NO, IL-1β, and TNF-α. In rats afflicted with EIU, the administration of ASPA resulted in a marked reduction of p-PI3Kp85 and p-Akt levels. Our findings point to ASPA’s role in curbing inflammation process in EIU, possibly mediated by the PI3K/Akt/NF-κB signaling cascade.

In conclusion, our findings indicate for the first time that ASPA has a mitigating effect on inflammation in LPS-induced EIU. Inhibition of the PI3K/Akt/NF-κB signaling by ASPA, as indicated by our data, is associated with a reduction in inflammation. These discoveries offer new insights into the relationship between ASPA and EIU, providing a foundation for further research on the molecular mechanisms and treatment of uveitis. Additionally, these results indicate that ASPA may emerge as a feasible option for addressing ocular inflammatory disorders.

## Data Availability

The datasets presented in this study can be found in online repositories. The names of the repository/repositories and accession number(s) can be found in the article/Supplementary material.
