# Supplementary material for: Asperulosidic acid inhibits the PI3K/Akt/NF-κB pathway to suppress endotoxin-induced uveitis
Source: Front Med (Lausanne). 2025 Jan 7;11:1524779. doi: 10.3389/fmed.2024.1524779 (PMC11746008; doi:10.3389/fmed.2024.1524779)
Supplement: Supplementary file 1 [file Data_Sheet_1.DOCX]

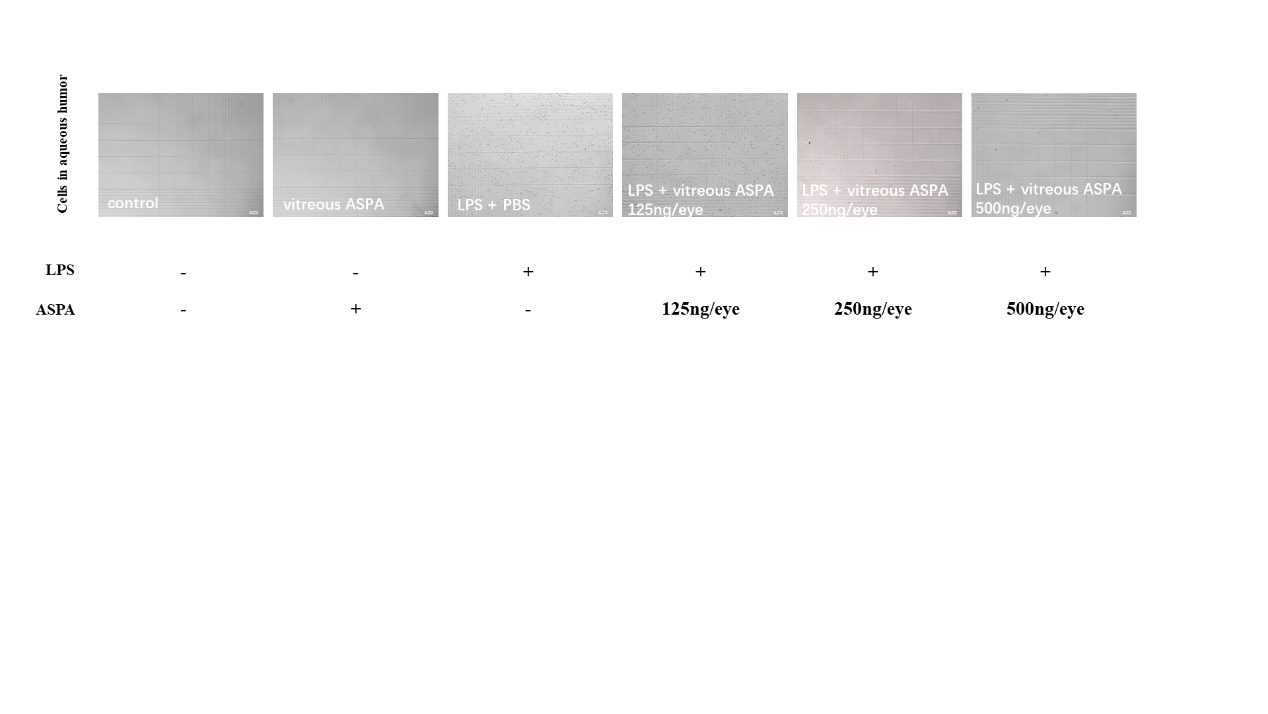


**Figure S1.** ASPA demonstrates a significant dose-dependent reduction in the quantity of inflammatory cells present in the anterior chamber of EIU rats.
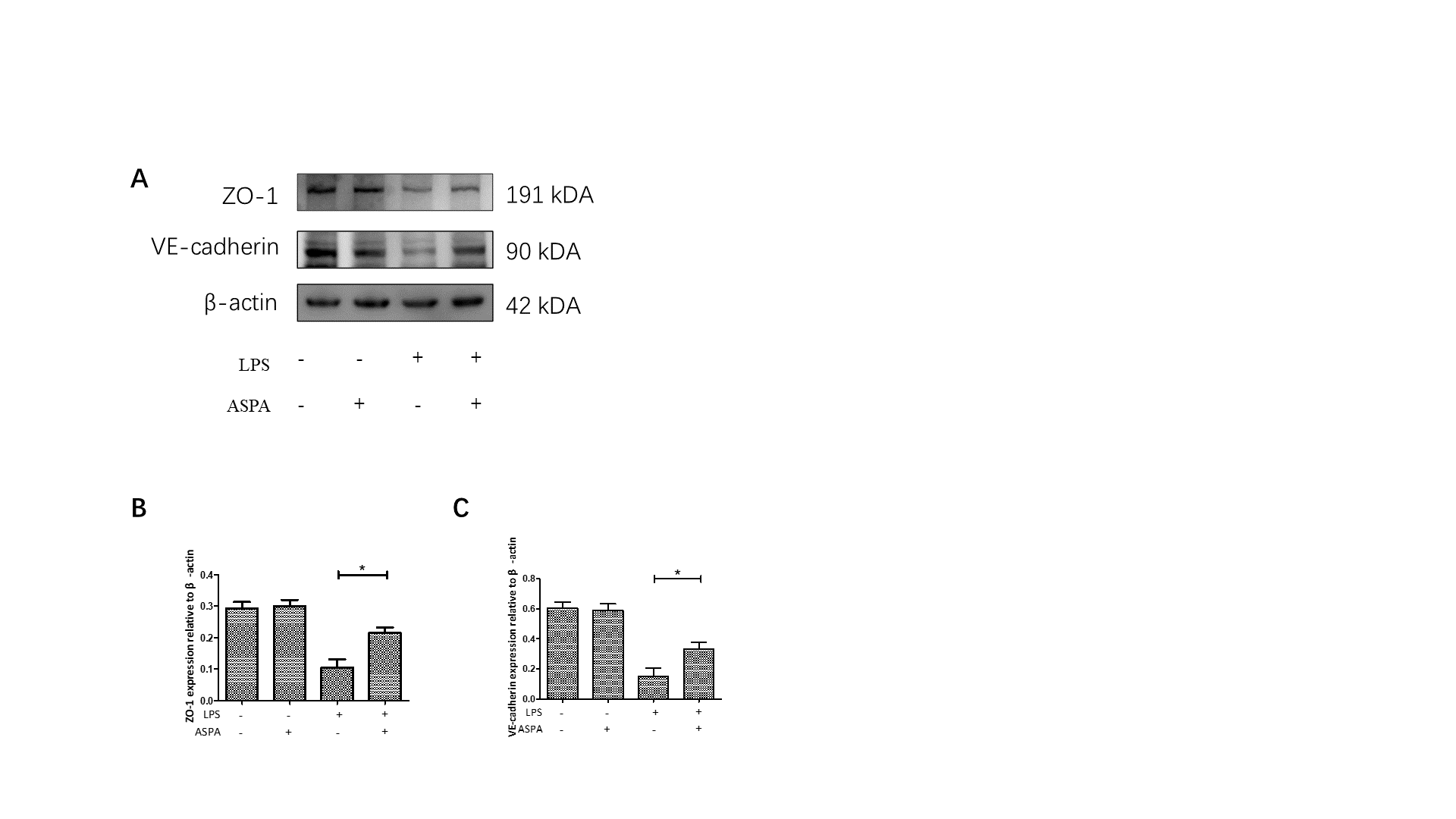


**Figure S2.** Western blot was used to detect the expression of ZO-1 and VE-cadherin in the retina of EIU rats (A). ASPA effectively counteracted the LPS-induced reduction in the expression of ZO-1(B) and VE-cadherin (C).
